# A qualitative interpretation of challenges associated with helping patients with multiple chronic diseases identify their goals

**DOI:** 10.15256/joc.2016.6.64

**Published:** 2016-11-14

**Authors:** Pauline Boeckxstaens, Sara Willems, Mieke Lanssens, Charlotte Decuypere, Guy Brusselle, Thomas Kühlein, Jan De Maeseneer, An De Sutter

**Affiliations:** ^1^Department of Family Medicine and Primary Healthcare, Ghent University, Ghent, Belgium; ^2^Department of Internal Medicine, Ghent University, Ghent, Belgium; ^3^Institute of General Medicine, University Clinic, Erlangen, Germany

**Keywords:** Multimorbidity, goal-oriented care, patient-centered care, primary care, family practice, qualitative research

## Abstract

**Background:**

Patients with multiple chronic diseases are usually treated according to disease-specific guidelines, with outcome measurements focusing mostly on biomedical indicators (e.g. blood sugar levels or lung function). However, for multimorbidity, a goal-oriented approach focusing on the goals defined by the individual patient, may be more suitable. Despite the clear theoretical and conceptual advantages of including patient-defined goals in clinical decision-making for multimorbidity, it is not clear how patients define their goals and which aspects play a role in the process of defining them.

**Objective:**

To explore goal-setting in patients with multimorbidity.

**Design:**

Qualitative analysis of interviews with 19 patients diagnosed with chronic obstructive pulmonary disease and comorbidities.

**Results:**

Patients do not naturally present their goals. Their goals are difficult to elicit, even when different interviewing techniques are used. Four underlying hypotheses which may explain this finding were identified from the interviews: (1) patients cannot identify with the concept of goal-setting; (2) goal-setting is reduced due to acceptation; (3) actual stressors predominate over personal goal-setting; and (4) patients may consider personal goals as selfish.

**Conclusions:**

Our findings advocate for specific attention to provider skills and strategies that help patients identify their personal goals. The hypotheses on why patients may struggle with defining goals may be useful to prompt patients in this process and support the development of a clinical method for goal-oriented care.

## Introduction

An aging population goes hand in hand with a rising prevalence of chronic diseases. More than half of patients aged over 65 years have two or more chronic diseases, and 20% have more than five [1]. Patients with multiple chronic conditions are usually treated according to disease-specific guidelines, with outcome measurements focusing mostly on biomedical indicators (e.g. blood sugar levels or lung function). These guidelines are based on evidence from classic randomized controlled clinical trials, which generally exclude patients with comorbid diseases. Consequently, disease management programs do not always meet the more complex needs of people with multimorbidity [2].

In 1991, Mold *et al*. challenged this disease-specific and problem-oriented approach in healthcare and proposed a paradigm shift towards goal-oriented care (Table 1), focusing on the patient’s individual goals instead of biomedical surrogate outcome measures [3]. Goal-oriented care could be tailored to the context of the individual patient and may be particularly suitable to support decision-making in the case of multimorbidity. However, despite the clear theoretical and conceptual advantages to include patient-defined goals in clinical decision-making for multimorbidity, it is not clear how patients define their goals and which aspects play a role in the process of defining them. Studies evaluating interventions that align with the concept of goal-oriented care often do not elaborate on the process of goal-setting [4–6]. Some authors report the use of skilled providers (goal facilitators [7]) to elicit patient goals, or have developed sophisticated tools for goal-setting [8–10]. In general, it is unclear whether providers have the basic skills to define patient goals or whether they need specific tool or skill sets to do so. Moreover, the search on useful strategies for goal-oriented care is complicated by the diverse terminology used in this field. This complicates the search and interpretation of relevant research findings. This qualitative study aims to explore the process of identifying patient goals through the use of different interview strategies and to identify possible underlying mechanisms on how patients define their goals.

## Materials and methods

### Study design

Considering the explorative nature of the research question, we adopted a qualitative study design based on patient interviews. All interviews were intended to elicit patients’ goals. Different interview strategies were used in different study phases. The analysis of the first phase informed the interview approach of the next phase. This paper reports on the overall analysis of three study phases. In the first phase (participants 1–7), an open-interview technique was used based on the Database of Individual Patients Experiences of illness (DIPEx) method [11]. The DIPEx group researches experiences of health and illness by interviewing people to explore what it is like to live with medical conditions. Through this approach, personal narratives inform the development of health services. We used this interview method to assess whether personal narratives would lead to the identification of personal goals. Interviewers did not use specific questions to elicit goals, but instead used their professional communication skills and theoretical background on goal-oriented care throughout the interview. The interview started with one open-ended question: *“Tell me what it is like to have COPD (chronic obstructive pulmonary disease) and other chronic conditions?”* The analysis of the first phase informed the second phase of the study. In this second phase (participants 8–13), a semi-structured approach was used. Questions were focused on the patients’ expectations of care (because respondents in the first phase tended to frame expectations in a disease-oriented framework) and on functional status using the components of the International Classification of Functioning and Disability in Health [12] (because the analysis of the narratives indicated that a biopsychosocial focus on functional status might bring us closer to patient goals). The analysis of this second set of interviews revealed that exploring the expectations of the family physician did not contribute to the clarification of the patient’s personal goals, whereas the discussion of functional status did provide useful information. Based on these new insights, the interview strategy was adjusted a second time. In a third phase (participants 14–19), the Canadian Occupational Performance Measure (COPM) was used as an interview guide [13]. This instrument has been developed and validated for goal-setting in occupational therapy. It provides a semi-structured interview setting in which patients first discuss their daily functioning and personal life. Consequently, the interviewer encourages and supports respondents to elicit and define personal goals by asking them to sum up five current priorities in their lives and define their personal goals for the forthcoming 5 years.

### Setting and sampling

Patients (*n*=19) with COPD who had at least one other co-existing chronic condition (comorbidity) were invited to participate in the study by their family physician or nurse.

The choice of an index disease when studying multiple chronic diseases can be argued. However, COPD was deliberately chosen. The disease-specific approach with inhalation therapy has shown little or no effect on mortality or deteriorating lung function [14]. The correlation between the biomedical outcome measure of lung function and patient’s functionality and quality of life is limited [15–17], and COPD patients often have comorbid diseases [18, 19]. Therefore, patients with multiple chronic diseases, including COPD, may be well suited to a shift from disease-oriented towards problem-oriented care.

After informed consent was obtained, the participants’ contact details were sent to the researchers. The inclusion of participants was a stepwise process and aimed at maximum variation (including both men and women of different age groups and backgrounds) [20]. Patients receiving palliative care and those with dementia were excluded from the study.

The study was approved by the Ethics Committee of the Ghent University Hospital, Ghent, Belgium.

### Data collection

Interviews were performed by primary care providers (family physicians [P.B., P.V.D.S., M.D.R., C.D.] and a registered nurse [M.L.]) with less than 5 years of clinical experience. These providers had been trained intensively in patient communication through their professional education. To avoid bias of any preceding insights into patients’ personal goals, none of the interviewers interviewed their own patients. All interviewers received basic training on qualitative interviewing, including an introduction to the theoretical concept of goal-oriented care as defined by Mold *et al.* [3] and Rueben and Tinetti [21]. These papers introduce goal-oriented care as a theoretical concept or paradigm, but not as a clinical method. This means that interviewers were not provided with any defined steps on how to apply goal-oriented care nor were they provided with a specific definition on what should be considered a patient goal. At the start of phase 3, P.B. and M.L. were additionally trained on the COPM [13] by an occupational therapist.

Interviews were performed between November 2008 and April 2013. Participants were contacted by telephone to set a time and date for the interview. Fifteen participants were interviewed in their home and four participants preferred to be interviewed in the practice setting. The average interview time was 65 minutes (range 30–99 minutes). All interviews were audiotaped and transcribed verbatim. Thirteen interviews were transcribed by the interviewer, and six interviews were transcribed by an administrative assistant. The patient quotes included in this manuscript were translated by an English teacher.

### Patient characteristics

The sample consisted of 19 participants (11 men and 8 women) with a mean age of 67 years (range 50–88 years). Twelve participants were married or in a relationship and living with their partner, seven participants were living alone, five were widowed, one was divorced, and one was living in a monastery. Participants had from three to 11 chronic diseases. The most recurring comorbid diseases were hypertension (*n*=9), osteoarthritis (*n*=6), a history of cancer (*n*=6), and reflux esophagitis (*n*=5). None of the participants with cancer were actively treated with chemotherapy or radiotherapy at the time of interviewing. The majority of participants were retired, two participants were still professionally active, and two were on sick leave (one permanently and one temporarily). Six participants were supported on a daily basis by a community nurse, eight participants received help for cleaning, and two participants received physical therapy. Four participants were in need of both a community nurse and professional help at home. One participant was institutionalized in a nursing home. Some participants also relied on the support of informal caregivers, including their children (*n*=4), partner (*n*=4), close relatives (*n*=1), or neighbors (*n*=1). In all cases, a family physician was involved in the care network. Fifteen participants were being actively treated or followed by a specialist (between one and five specialists per participant). Pneumologists (*n*=13) and cardiologists (*n*=5) were primarily involved in the care of these patients with COPD and comorbidities.

### Data selection, coding, and analysis

All transcripts were carefully read several times by both P.B. and M.L. This reading confirmed the fact that the process of goal-setting had been difficult throughout all phases of the study. All transcripts were screened for participant quotes, which were considered relevant to explore the process of goal-setting. The selection of relevant quotes was conducted independently by P.B. and M.L. After each set of three interviews, both researchers discussed their selection. All participant quotes that were considered to be potentially relevant by at least one researcher were included for further analysis.

The participant quotes that were selected were then analyzed independently by P.B. and M.L. using open coding. Regular meetings were held to discuss the codes. The initial findings were then presented to A.D.S., an expert in goal-oriented care, and to S.W., an expert in qualitative research and a senior researcher in primary care topics. This presentation was followed by extensive team discussions leading to an in-depth interpretation of the data.

## Results

### Identifying personal goals

Despite the different strategies to support participants to define their personal goals, the main finding of this study was that even with the use of COPM (an instrument specifically developed and validated for this purpose), goals were difficult to define and express. When a patient defined a goal, it was often expressed in a very general way. *“Being healthy is my first priority” (Participant 8). “Being healthy” (Participant 11). “What is most important is that my partner’s health would be as good as it gets. And that the people I love are well, that they are happy” (Participant 14).*

Respondents stated that they wanted their current health status or situation to remain “*the way it is now*”, as a main goal. They focused on no further deterioration, but again, expressed this in a very general way. *“To feel the way I feel now” (Participant 1). “It can stay like this for a long time, as long as it does not get worse” (Participant 17). “I wish it would stay as it is now” (Participant 19)*. Only a limited number of participants defined specific goals, which they wanted to maintain. Many of these were defined at the level of participation in society. *“To go out to the seaside and have a walk and look at the shops” (Participant 6). “To travel once in a while, to be able to sail” (Participant 16). “I would still like to do some sports” (Participant 18)*. Participants with more severe disease tended to formulate goals at the level of activities of daily living. *“To leave the house and enjoy myself” (Participant 1). “To go to the toilet independently” (Participant 7)*. Only one participant formulated a goal, which extended beyond his current level of activity. This patient was still recovering after a long episode on intensive care due to a complicated appendicitis. *“Maybe this summer, since I feel so much better, I can mow the lawn myself” (Participant 6)*.

### What makes it so difficult to identify personal goals?

Departing from the data, four hypotheses were developed to explain why patient goals might be so difficult to identify. First, patients could not identify with the concept of goal-setting. Even in the most structured interviews where the COPM method was used, participants did not seem to know what we meant by “goals” or “goal-setting”. They did not understand or even relate to the concept. Interviewer:* “So we have discussed a lot of things now. Say we would be able to look 5 years ahead, what would you like to achieve or what would you want to maintain? What are the things that matter the most to you?”* Respondent:* “Well, that I could be like my mother. She was 90 years old and she didn’t have a family doctor and she didn’t take any pills. She lived until she was 94! And she only started to need a doctor when she was 90 because her heart got weaker”*. Interviewer:* “So, what would make you say, I have achieved this?”* Respondent:* Well, that it can stay like this, I don’t know, … with medication probably… I don’t know”.* Interviewer:* “And how would you be able to stay like this, what or who could help you to achieve this?”* Respondent:* “Well, going to the doctor. It’s not something I am able to do” (Participant 8).*

Some patients seem to have accepted their situation and do not feel the need to set goals. *“I feel happy now” (Participant 2). “If you accept it, you feel happy. If you don’t accept it, you feel unhappy” (Participant 3). “I’m satisfied with how I am at this moment” (Participant 4)*. Age seemed to foster acceptance and minimized goal-setting. *“I have lived my life” (Participant 3). “My time is passed” (Participant 19)*. The nature of chronic diseases may also influence acceptance. Because they have no cure, patients may feel like they have no choice but to accept their illnesses. Moreover, because most chronic diseases have a slow but progressive course, patients accept their situation over time.* “My health can’t improve, I have to accept that. I do accept that as much as I can” (Participant 12). “After some time, you get used to it” (Participant 17).* Some participants actually described a reduced need for social participation. They seemed to have gradually cut down on social activities and no longer felt the need to engage in the community. *“I used to enjoy asking people over for a visit. I thought this was fun and joyful, but now it is a burden” (Participant 1). “Now I enjoy peace and quiet. Before I didn’t, I would have been a nervous wreck if I had to stay home for one day” (Participant 5).* Despite the observation that patients seemed to reconcile with shrinking social networks, this might not always be true. *“I accept my fate. … I don’t like it that I watch TV all day” (Participant 1). “I say we have to learn to live with it… Sure it is difficult for me to accept that. I am often angry with myself” (Participant 2). “There is nothing you can do about it, you just have to deal with it…. That has always been a battle” (Participant 19).*

Some participants described stressors such as pain, fear, or exhaustion. These stressors might feature so prominently that the goal-setting process fades into the background. Patients switched to “survival mode”. *“So I live with pain, constant pain” (P10).* Fear was a much-discussed subject throughout most interviews and was not merely related to COPD. *“I am afraid of suffering” (Participant 1). “My biggest fear is that I will be admitted to the hospital. That I won’t return home” (Participant 12). “Sometimes I have panic attacks. Afraid of dying of cancer or something else that goes wrong” (Participant 15).* Despite the fact that most participants were rather optimistic regarding their functional status, some reported that they lack the physical and/or psychological capacity to perform certain activities. They want to, but they cannot. They felt a need to pace their life rhythm. *“I have been in the hospital for 18 months, my lungs are healed, but my mental health is shot” (Participant 14). “Some days I feel strong, but then other days I have to lay down on the couch. That wears you down” (Participant 19).*

Being dependent on others was described as one of the main problems of having COPD and comorbidities. Patients felt like they were bothering others and tried to avoid asking for help as much as possible. *“The annoying part is that you always have to bother someone else” (Participant 4).* Participants tried to diminish the burden for the healthcare workers and even support them. Consequently, participants might feel uncomfortable defining personal goals because they place others’ concerns before theirs. *“I make sure everything is set when the nurse comes” (Participant 4). “I want my wife to continue to do what she likes most” (Participant 9). “My daughter is the major victim, she has to do everything” (Participant 15).*

## Discussion

In this study, we found that respondents with COPD and comorbidity do not naturally present their goals. Both narrative and more structured interview approaches did not result in a clear list of patient goals. This is an interesting observation, as many authors who publish on goal-oriented care seem to consider the identification of goals to be a matter of course. Only a few authors have reported in detail on the process of identifying patient goals. Kuluski *et al*. [22] identified patient goals through a DIPEx-inspired semi-structured interview that ended with the open-ended question: *“Do you have care goals? In other words, what would you say are your most important goals for doing the things you want to do, staying in the best health you can attain and living the life you believe you can live?”* The authors of this study did not report any challenges on having these questions answered. Purkaple *et al*. [23] reported fairly good response rates from patients with the use of written answers for three questions that may relate to goals: (1)* “What is a typical day for you and what things are you unable to do as a result of your health problems?”*; (2)* “What other things would you like to be able to do that you can’t do now?”*; and (3) *“What activities make life worthwhile for you (that you wouldn’t want to give up?)”*. This approach is in line with the approach we have used in the third phase of our study. It is possible that a written approach may leave more room for personal reflection and may provide a more straightforward approach than an interview, which can be prone to flawed and confusing questions.

To our knowledge, this is the first study to explicitly identify that patients do not naturally present their personal goals to providers. Despite obstacles at the provider level, our results also indicated barriers at the patient level. Whether this is because patients actively reconcile to their present situation (in other words, do not feel the need to set goals) or whether they just give up setting goals (because they think they cannot attain them) is unclear. Other barriers, such as dominating stressors (such as pain, fear, or exhaustion), or placing other’s concerns over their own, may also impede goal-setting in patients. Moreover, despite the theoretical advantages of goal-oriented care for people with multimorbidity, this study cannot confirm that patients need or expect goal-oriented care. This may question the concept of goal-oriented care in itself. However, patients may just not be ready to define personal goals. Steele Gray *et al*. [9, 10] have specifically accounted for this in the development of their electronic patient-reported outcome tool. If a patient is not ready, patient-reported outcomes can be assessed without prioritization by the patient. The authors hypothesize that this, in itself, may be supportive of the goal-setting process. In the same context of patient readiness, Tinetti *et al*. [7] hypothesize that individuals who are experiencing increased healthcare utilization or who regret treatment decisions (such as undergoing a procedure), may be particularly ready to consider personal goals and priorities. Considering this, patients may better express goals in relation to specific scenarios. However, provider-defined scenarios may be prone to an overemphasis of providers’ perspectives. Therefore, this study intentionally approached patients in a context unrelated to clinical decision-making and did not work with predefined types of goals that had to be identified through the patient interviews. An additional barrier to patients reporting clearly on their personal goals could be that healthcare systems shape patients’ perspectives and expectations towards care. Patients might not be used to providers focusing on patient-centered outcomes, and may conform to a biomedical and problem-oriented approach. They may not be able to imagine that they can introduce their personal goals into clinical decisions. Reuben and Tinetti [21] defined medicine to be too deeply rooted in a *“disease-outcome-based paradigm”* as the most important barrier to goal-oriented care. This hypothesis has actually been ratified recently through the paper of Purkaple *et al*. [23] who found that the goal-related issues identified by patients were only mentioned in two out of 64 primary care encounters. In neither case was this information used in clinical decision-making.

Even though more specific training of interviewers or more specific strategies of identifying personal goals of patients may have yielded better results, this open and non-specific approach to patients should be considered a strength. This approach clarifies the importance of paying specific attention to the process of goal identification. The fact that the interviewers did not know the patient beforehand may be a limitation, as a continuous relationship between the provider and the patient may have facilitated the process of identifying personal goals. Another limitation is that the participants were not engaged in the interpretation of the data [24]. Patients themselves could have increased our insight into the phenomenon of personal goal-setting and might have countered any possible over-interpretation of the results (both at the levels of goal identification and definition of the hypotheses).

Even though our observations may have been caused by a flawed technique or the wrong approach to interviewing patients, this study can conclude that thorough training in patient communication and a theoretical background on the concept of goal-oriented care are not sufficient to elicit patients’ personal goals. Providers should be trained specifically in how to explain the concept of goal-setting to patients and how to support them to overcome barriers to goal-setting (such as over-acceptance, actual stressors, and fear of being selfish). Talking about patient goals in the context of a continuous relationship between the provider and the patient may facilitate the process of defining patient goals. Another strategy may be to talk about patient goals in the context of a specific scenario or within a health encounter. However, other authors did encounter diverging priorities between patients and providers in this context [25]. Prior preparation of patients through the use of goal-oriented questionnaires may be a strategy less prone to overemphasis on provider and health system goals. These hypotheses should be addressed through further work.

The eventual objective in clinical practice is to accomplish collaborative goal-setting between patients and providers. If patients are unable to identify their personal goals, collaborative goal-setting processes in clinical encounters may tend to over-emphasize provider or health system goals. Our results increase awareness of the importance of the process of identifying personal goals. In the broader context of goal-oriented care, these results indicate a need to develop the theoretical concept of goal-oriented care into a clinical method. Compared with a theoretical concept, a clinical method defines specific steps on how to apply the concept and develops measurement and teaching tools. The patient-centered clinical method can be a useful guide in the process of developing goal-oriented care for people with multimorbidity from a theoretical concept into a clinical method [26].

Further work in this field should engage patients from the onset of the study and on an ongoing basis in order to merge their insights and perspectives with the scientific and clinical knowledge of an extended research team. This team should include experts in the patient-centered clinical method, sociology, occupational therapy, and others [27]. At the policy level, the healthcare system should become more attentive to goal-oriented care, instead of problem-oriented care, in order to support providers in fully engaging in the process of goal-oriented care. The current focus on fee for (technical) services and quality assessment through disease-oriented outcome and process measures is not well suited to a goal-oriented approach in healthcare.

## Conclusions

One approach to improving care for people with multiple chronic diseases is for healthcare providers to refocus care from treating individual diseases in isolation to achieving people’s personal goals. However, it is unclear how patient goals can be elicited and who should do this. This study illustrates that patients do not naturally present their goals to providers, and indicates that specific attention needs to be paid to the process of identifying patient goals.

## Figures and Tables

**Table 1:** Problem-oriented versus goal-oriented care [3].

	Problem-oriented care	Goal-oriented care
Definition of health	Absence of disease as defined by the healthcare system	Maximum desirable and achievable quality and/or quantity of life as defined by each individual
Purposes of healthcare	Eradication of disease, and prevention of death	Assistance in achieving a maximum individual health potential
Measures of success	Accuracy of diagnosis, appropriateness of treatment, eradication of disease, and prevention of death	Achievement of individual goals
Evaluator of success	Physician	Patient
